# Supplementary material for: Exploring the Therapeutic Potential of the DOT1L Inhibitor EPZ004777 Using Bioinformatics and Molecular Docking Approaches in Acute Myeloid Leukemia
Source: Curr Issues Mol Biol. 2025 Mar 4;47(3):173. doi: 10.3390/cimb47030173 (PMC11941229; doi:10.3390/cimb47030173)
Supplement: Supplementary file 1 [file cimb-47-00173-s001.zip › cimb-3476663-supplementary.pdf]

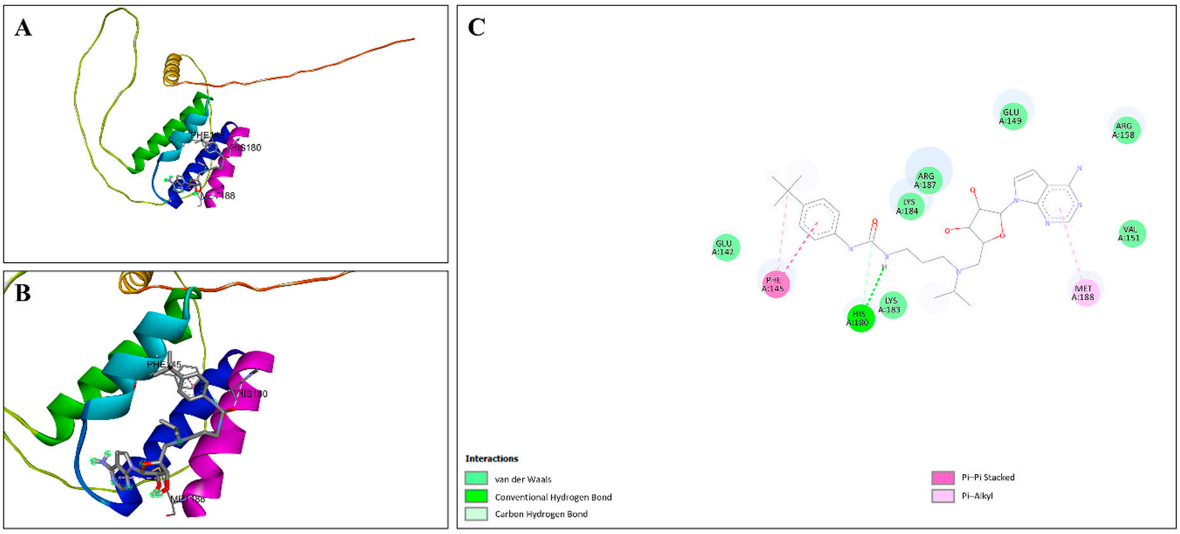

Figure S1. Molecular docking analysis of EPZ004777 with CT45A3. (A) Docking pose of EPZ004777 within the CT45A3 binding site, showing its position on the protein surface with secondary structural elements. **(B)** Close-up view of specific interactions between EPZ004777 and CT45A3. **(C)** 2D interaction diagram depicting hydrogen bonds, π–π stacking, Pi–Alkyl interactions, and van der Waals forces, illustrating the stabilizing network within the active site.

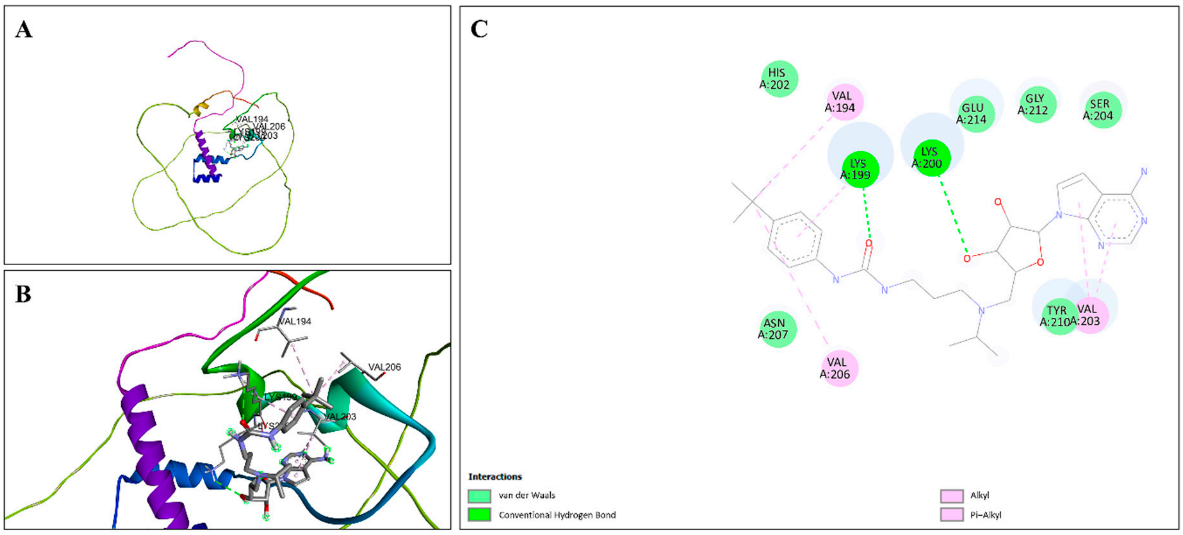

Figure S2. Molecular docking analysis of EPZ004447 with HOXA4. (A) Docking pose of EPZ004447 within the HOXA4 binding site, showing its position in the pocket with secondary structural elements. **(B)** Close-up view of specific interactions between EPZ004447 and HOXA4. **(C)** 2D interaction diagram illustrating hydrogen bonds, van

der Waals forces, and hydrophobic interactions such as alkyl and Pi–Alkyl interactions, demonstrating the stabilizing network within the binding site.

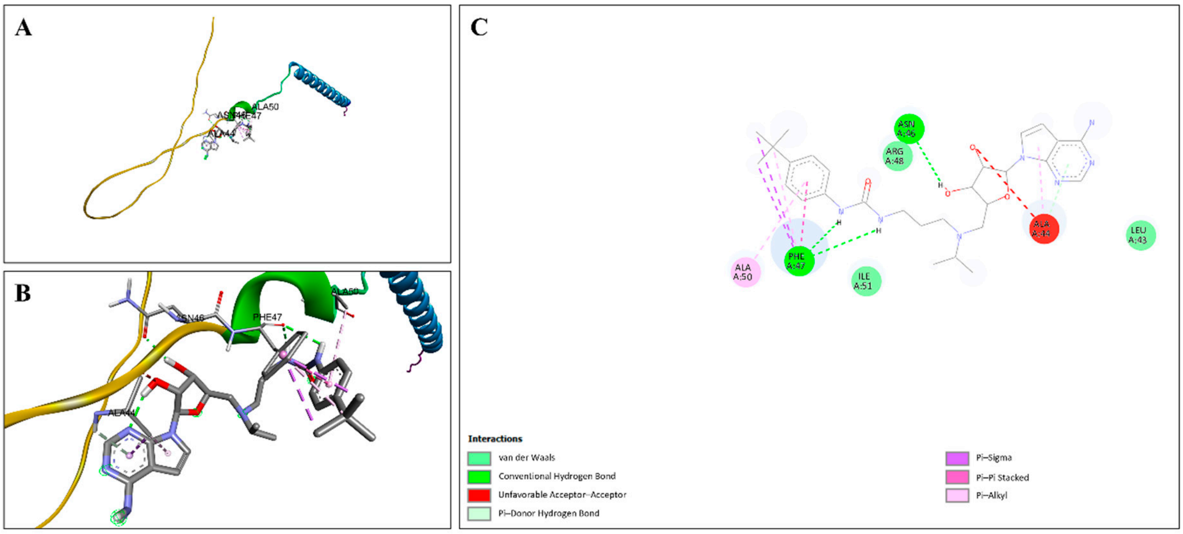

Figure S3. Molecular docking analysis of EPZ004777 with BEX3. (A) Docking pose of EPZ004777 within the BEX3 binding site, showing its position on the protein surface with secondary structural elements. **(B)** Close-up view of specific interactions between EPZ004777 and BEX3. **(C)** 2D interaction diagram illustrating hydrogen bonds, π–π stacking, and unfavorable acceptor–acceptor interactions, along with van der Waals forces, highlighting the stabilizing network within the binding site.
